# Exercise-induced popliteal artery compression due to a popliteal fossa cyst

**DOI:** 10.1016/j.jvscit.2026.102305

**Published:** 2026-05-14

**Authors:** Vidalack Vilayphone, Zhixiong Zhang, Rongwen Xiao, Xiaojiong Du, Yi Yang

**Affiliations:** a Division of Vascular Surger, Department of General Surgery, West China Hospital, Sichuan University, Chengdu, China; b Department of Ultrasound Medicine, West China Hospital, Sichuan University, Chengdu, China

**Keywords:** Popliteal fossa cyst, Baker cyst, Popliteal artery stenosis, Intermittent claudication, Popliteal artery compression

## Abstract

Nonatherosclerotic extrinsic compression from para-articular cysts is a rare but clinically important cause of claudication. We report the case of a 50-year-old man who presented with left calf claudication and absence of distal left dorsalis pedis artery pulse after exertion. A duplex ultrasound study revealed a popliteal fossa cyst that doubled in size under exertion (from 3.2 to 7.2 mm), leading to an extrinsic compression of the popliteal artery. Magnetic resonance imaging scans confirmed a possible articular origin of the cyst. Dense adhesions surrounding the lesion led to the reconstruction of both the left popliteal artery and left popliteal vein. This case highlights the importance of implementing exercise during duplex ultrasound study of lower limbs to identify rare exercise-induced enlargement of popliteal cysts causing popliteal artery stenosis, which may subsequently require complex multivessel surgical management.

Intermittent claudication, defined as exertional lower limb muscle pain relieved by rest, is a typical clinical indication of peripheral artery disease, with over 95% of cases being caused by atherosclerosis.^1^ Rare pathologies contributing to popliteal artery stenosis (PAS) in patients without typical risk factors include popliteal artery entrapment syndrome, which resulted from anomalous musculotendinous compression of the popliteal artery (PA),^2^ and cystic adventitial disease (CAD), caused by accumulation of gelatinous mucoproteins within the adventitia area, resulting in arterial compression.^3^ An even rarer cause is extrinsic compression by adjacent masses, such as popliteal fossa cysts (Baker cysts) or other benign/malignant tumors.^4^ Although Baker cysts are common, they rarely cause mechanical compression leading to ischemia.

We report a complex case of a 50-year-old man with intermittent claudication due to persistent compression from an enlarged popliteal fossa cyst. Duplex ultrasound and contrast-enhanced ultrasound scans were performed to evaluate the severity of the suspected PAS. Surgical management required reconstruction of both the left popliteal artery (LPA) and left popliteal vein (LPV). This case highlights the association between knee joint pathology and vascular compression, addressing a rare occurrence of surgical management with such multivessel involvement. Written informed consent was obtained from the patient for publication of this case report and the accompanying images.

## Case Presentation

The patient was a 50-year-old man who presented to the vascular surgery outpatient clinic with a 3-month chief complaint of intermittent claudication in his left calf. Symptoms were reproducible, and walking distance was limited to approximately 1000 m. The patient had a known history of hypertension for over 10 years and denied any history of smoking, diabetes, or coronary artery disease. Magnetic resonance imaging (MRI) confirmed the presence of a multiloculated cyst in the left popliteal fossa and revealed underlying knee joint arthritis, along with a likely medial meniscal tear and possible anterior cruciate ligament and posterior cruciate ligament injuries. Focal bone marrow edema was also observed on MRI, suggesting an articular origin of the cyst.

Duplex ultrasound study of the lower limbs was performed when the patient was at rest and after exercise. At rest, pulses of the left dorsalis pedis artery and posterior tibial artery were palpably normal. The patient was instructed to walk briskly back and forth along a flat corridor for 5 min/cycle, totaling three cycles (15 minutes total) of ambulation, with only minimal rest between cycles. Following the three cycles of exercise, pulse of the dorsalis pedis artery became nonpalpable. Duplex ultrasound study was conducted using a Philips ultrasound system with a 9 MHz linear transducer to evaluate flow limitation. A left lower limb ultrasound scan was performed between cycles, with the patient standing before the exercise. It revealed an anechoic, multiloculated cystic mass anterolaterally, measuring 25 × 13 mm in cross-section, and posteriorly, 3.2 mm in diameter, found engulfing the LPA.

**Fig 1:**
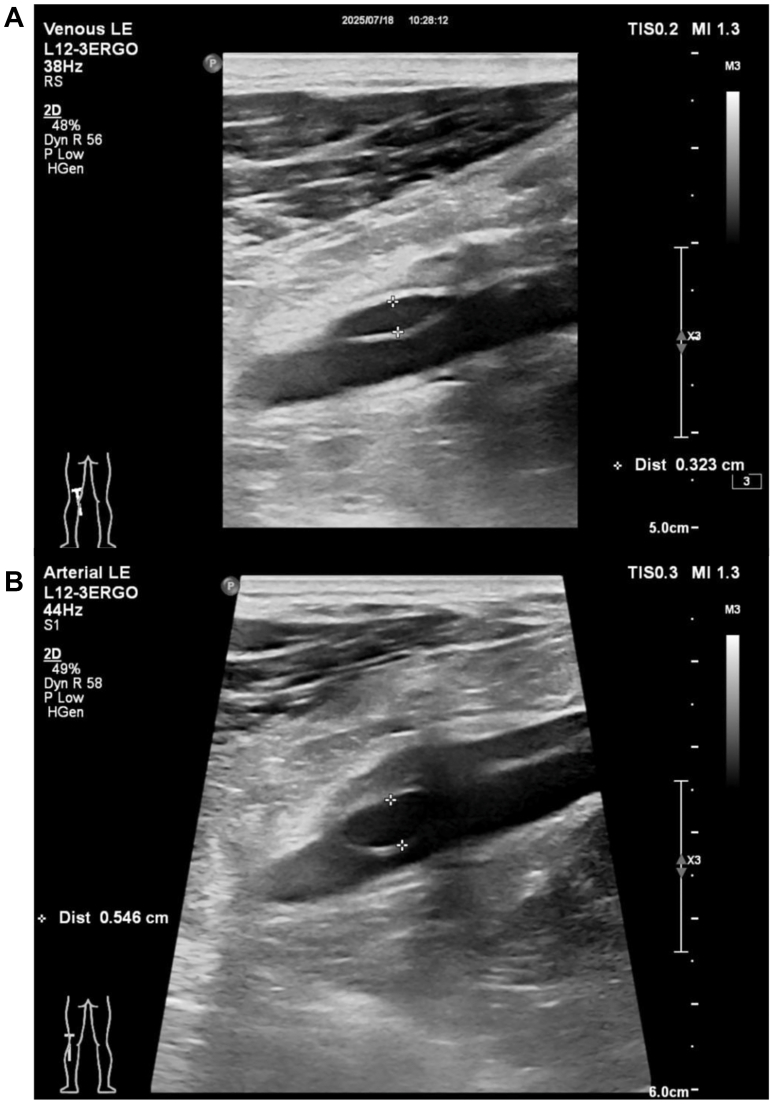
**(A)** At rest, the diameter of the popliteal artery was 4 mm, and the diameter of the posterior popliteal cyst was 3.2 mm (+). **(B)** After the second exercise cycle, the popliteal fossa cyst located posterior to the popliteal artery was measured 5.5 mm in diameter, resulting in popliteal artery compression.

**Fig 2:**
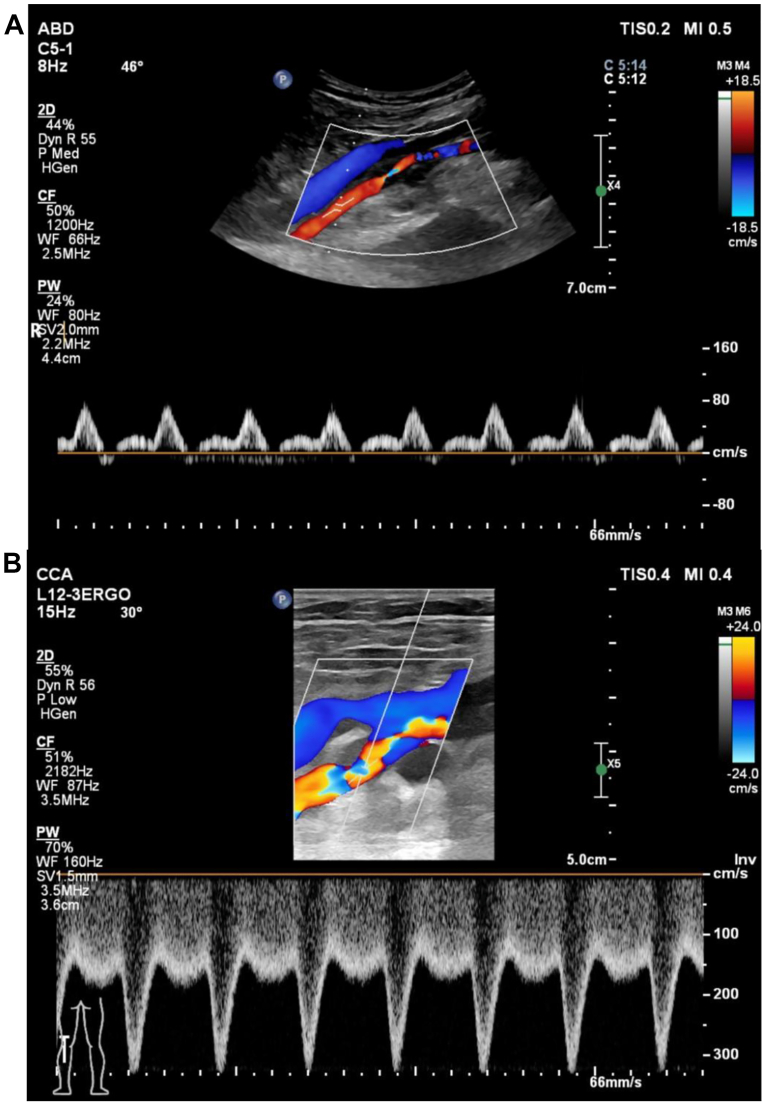
**(A)** The Doppler ultrasound scan showed the peak systolic velocity (PSV) of the proximal segment of the popliteal artery at the site of compression was less than 80 cm/s. **(B)** The Doppler ultrasound scan indicated that the blood flow velocity at the site where the popliteal artery was compressed increased, with the PSV exceeding 300 cm/s, although the standard criteria define a PSV over 275-300 cm/s as indicative of severe stenosis, typically 70%-99% narrowing.

**Fig 3:**
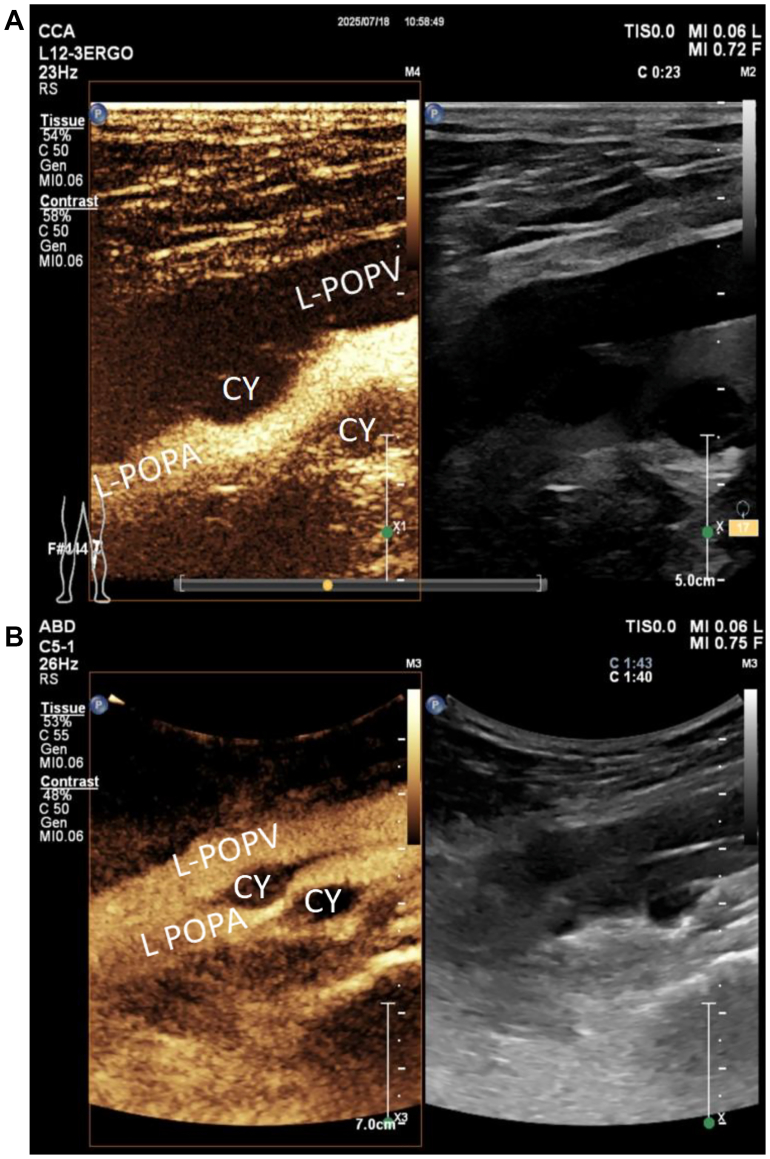
**(A)** Contrast-enhanced ultrasound (CEUS) scan from the first exercise cycle showed the left popliteal artery (*L-POPA*) was compressed by the popliteal cyst (*CY*) during the arterial phase, resulting in mild stenosis of the lumen. **(B)** CEUS scan from the third exercise cycle showed that the popliteal artery was compressed by the popliteal cyst, resulting in severe stenosis of the lumen during the venous phase. *L-POPV*, Left popliteal vein.

After the second exercise cycle, the ultrasound study demonstrated enlargement of the posterior popliteal fossa cyst from 3.2 to 5.5 mm (Fig 1, *A* and *B*), causing severe stenosis of the LPA as observed on Doppler ultrasound examination (Fig 2, *A* and *B*). The ultrasound scan measured the cyst up to 7.2 mm in diameter after the third exercise cycle. Subsequently, a contrast-enhanced ultrasound study confirmed 70% stenosis of the PA due to the popliteal fossa cyst, with the narrowest luminal diameter measuring 2.1 mm, compared with a distal vessel diameter of 7 mm (Fig 3, *A* and *B*).

**Fig 4:**
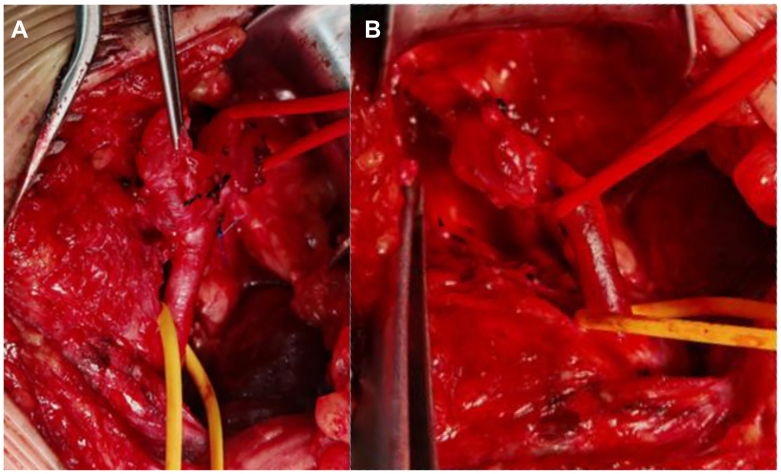
The intraoperative view shows that the cyst was strongly adhered anterolaterally **(A)** and posteriorly **(B)** to the popliteal artery.

**Fig 5:**
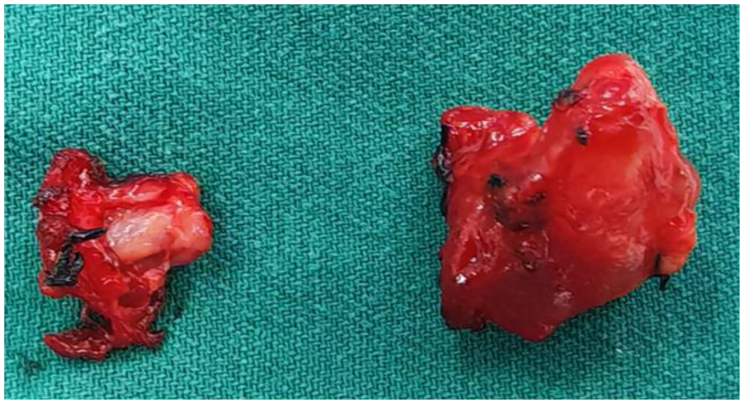
Resected surgical specimen submitted for histopathological analysis, confirming the presence of synovial lining with adhered arterial tissue.

Based on these findings, the patient underwent cyst excision surgery. The patient was placed in the prone position, and an open posterior popliteal approach was used through an S-shaped incision centered over the popliteal fossa, extending across the popliteal crease to provide wide exposure. Intraoperatively, a 3 × 2 × 2 cm mucoid mass was identified posterolateral to the PA (Fig 4, *A* and *B*). The apparent difference from the preoperative ultrasound description (anterolateral) was attributed to patient positioning and imaging perspective. The cyst wall was densely adherent to both the LPA and the LPV. The inability to safely separate the cyst from the vessel necessitated the resection of the LPA and LPV, followed by subsequent reconstruction. The histopathology report confirmed a popliteal fossa cyst with the arterial wall tissue adherent to the excised specimen (Fig 5). The patient had an uneventful postoperative recovery and reported complete resolution of claudication by 4 weeks. Routine follow-up appointments were scheduled to monitor for any future complications.

## Discussion

This case demonstrates that nonatherosclerotic peripheral artery disease can also be attributed entirely to mechanical factors. It represents a classic case of exercise-induced flow limitation, which is commonly observed in mechanical compression conditions such as CAD or popliteal artery entrapment syndrome.^5^ The patient's presentation was clinically indistinguishable from classic CAD, as evidenced by a normal resting pulse that became nonpalpable after exertion. The histopathologic findings clarified the distinction between a Baker cyst and CAD. A true Baker cyst exhibits a synovial membrane lining,^6^ whereas CAD involves mucin-filled cysts within the arterial wall adventitia, without a true synovial lining.^5^ In the present specimen, the presence of synovial lining and entrapped arterial tissue confirmed the diagnosis of a Baker cyst origin despite its deep location. Percutaneous aspiration was avoided because of the high recurrence rates.^7^ Its intimate involvement with adventitia mimicked CAD intraoperatively, requiring both PA and popliteal vein reconstruction. This finding further underscores the complexity and multivessel involvement when managing with densely fibrotic or inflammatory masses.^8^

Essential diagnostic differentiation involves recognizing the condition as mechanical compression syndrome and performing duplex ultrasound imaging to calculate the blood flow limitation.^9^ Such exercise-induced flow changes are characteristic of arterial compression syndromes. Ultrasound imaging and the histopathology report ruled out CAD and confirmed a diagnosis of popliteal fossa cyst. Implementing exercise during ultrasound scanning is therefore mandatory for evaluating claudication, especially in the presence of popliteal fossa masses, regardless of patient age.

The pathophysiology appears linked to joint effusion. MRI findings of knee arthritis and meniscal tear support the ganglion/articular theory.^10^ During physical movement, the increased hydraulic pressure within the damaged knee joint may force synovial fluid through a one-way valve into the cyst.^11^ This rapid fluid effusion leads to an acute elevation of intracystic pressure and volume,^11^^,^^12^ causing rapid enlargement (from 3.2 to 7.2 mm) with exertion, suddenly impinging the PA. Given the MRI evidence of underlying joint pathology, cyst recurrence remains a concern unless the knee lesions are addressed.^13^ In this case, the patient was subsequently referred to the orthopedic department for further management of the meniscal tear and underlying joint arthritis to minimize the risk of recurrence.

## Conclusion

This case report documents a rare presentation of severe exercise-induced PAS secondary to an extrinsic popliteal fossa cyst. PAS due to popliteal fossa cysts should be considered in patients with claudication and minimal atherosclerotic risk factors. As Baker cysts are associated with joint disease, clinicians should also address underlying rheumatic pathology to reduce the risk of recurrence.^12^ Importantly, using exercise during vascular imaging is essential for differentiating functional causes of claudication and unmasking possible vascular compression, thereby preventing potential long-term arterial damage from repetitive microtrauma.

## Declaration of generative AI and AI-assisted technologies in the writing process

During the preparation of this work, the authors used Gemini to enhance academic English vocabulary and improve the manuscript content to meet academic standards. After using this tool, the authors reviewed and edited the content as needed and take full responsibility for the content of the publication.

## Funding

No funding was provided.

## Disclosures

The authors have no competing interests.
